# Approach to Different Types of Inferior Vena Cava Leiomyosarcomas: A Vascular Surgeon’s Perspective

**DOI:** 10.7759/cureus.40694

**Published:** 2023-06-20

**Authors:** Rajeev Thilak Chellasamy, Ananthkumar Sivanesan, Aravind Kalyanasundaram, Hemachandren Munusamy, Nembian Rajarajan

**Affiliations:** 1 Cardiothoracic and Vascular Surgery, Jawaharlal Institute of Postgraduate Medical Education and Research, Puducherry, IND

**Keywords:** vascular reconstruction, complex surgical resection, tumors, vascular leiomyosarcoma, inferior vena cava

## Abstract

Leiomyosarcoma (LMS) is a rare smooth muscle tumor, and only a few cases have been reported with involvement of the inferior vena cava (IVC). Inferior vena cava LMS is more often silent and usually has a poor prognosis as the patients present late. We present this case series to showcase the different approaches to surgical resection, as each tumor had a different location in the IVC. We emphasize preoperative surgical planning to achieve a tumor-free margin and maintain hemodynamics at the same time.

## Introduction

Leiomyosarcomas (LMS) are very rare tumors that arise from smooth muscle cells. They originate from the tunica media when they involve vessels. The incidence of inferior vena cava (IVC) LMS is 0.5% of all adult tissue sarcomas [1]. It is more common in the sixth decade of life and is more prevalent in women [2]. The survival rate improves after complete surgical resection of the tumor with negative margins [3]. We report three cases of IVC LMS, with different locations in each patient. A 28-year-old female patient was incidentally found to have a mass arising from the posterior wall of the IVC. The second patient was a 35-year-old female with sub-hepatic IVC LMS who presented with non-specific abdomen pain with pedal edema. Finally, a 30-year-old female presented with a suprahepatic IVC tumor. Surgical resection was done for all the patients, and all postoperative biopsies were suggestive of IVC LMS. We present this report to highlight the different surgical approaches taken due to the varying locations of the tumors in each patient.

## Case presentation

Case 1

**Figure 1:**
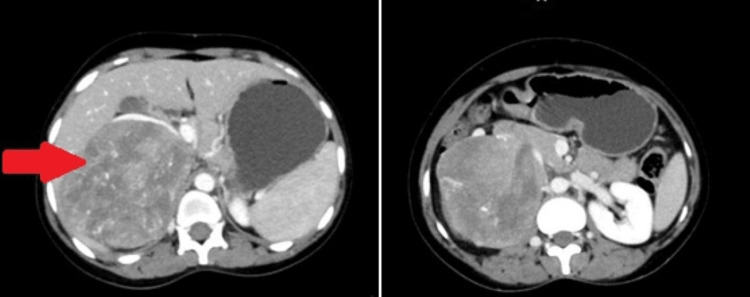
CECT of the abdomen with the red arrow pointing to the large IVC tumor CECT: Contrast-enhanced computed tomography, IVC: Inferior vena cava

A 28-year-old female patient was incidentally found to have a supra-renal mass when she underwent routine ultrasound for non-specific complaints. Contrast-enhanced computed tomography (CECT) of the abdomen showed a mass arising from the posterior wall of the IVC (Figure 1). The patient was taken up for surgery in view of the size of the mass. The tumor was resected along with the posterior wall, and primary repair of the IVC was done.

Case 2

**Figure 2:**
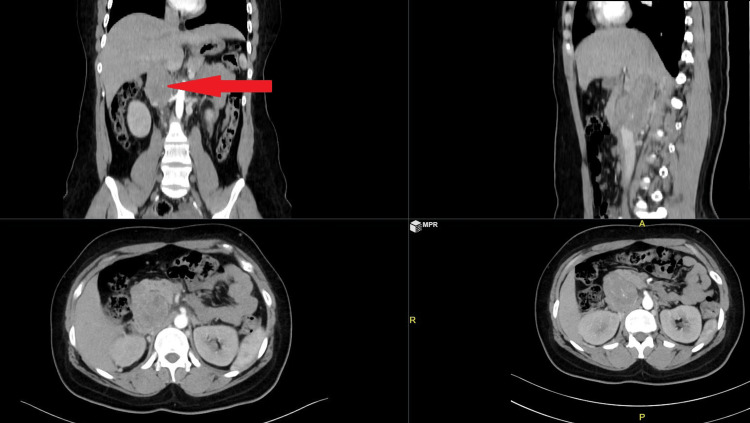
CT abdomen with sub-hepatic mass marked by a red arrow

**Figure 3:**
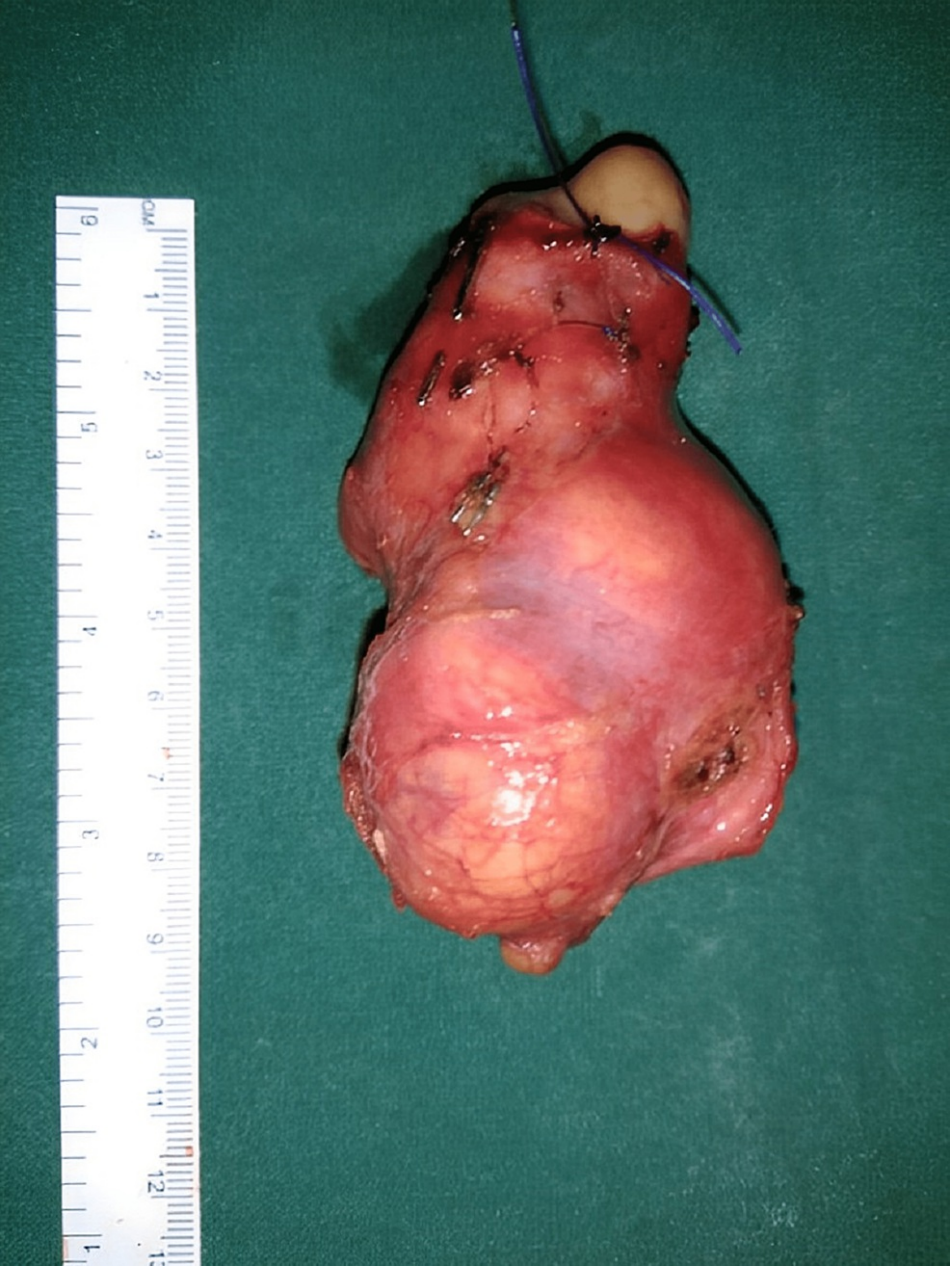
Resected specimen along with IVC IVC: Inferior vena cava

**Figure 4:**
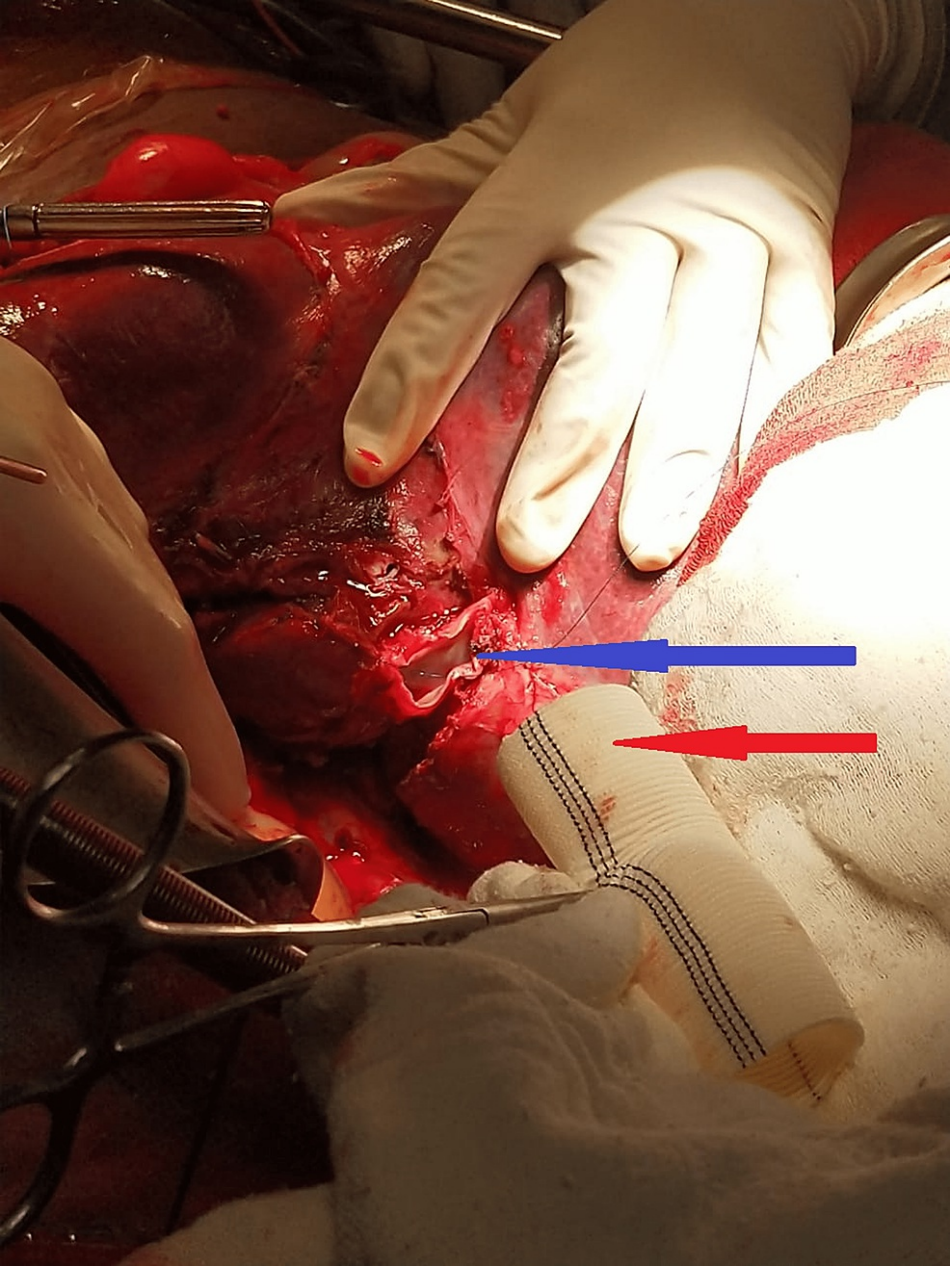
Intraoperative image with the blue arrow marking the resected end of the IVC and red arrow pointing to the Dacron graft IVC: Inferior vena cava

A 35-year-old female patient presented with non-specific abdominal pain with pedal edema. On evaluation, she was found to have a sub-hepatic mass involving the IVC on CT of the abdomen (Figure 2). Mass was resected along with the IVC (Figure 3), and IVC reconstruction was done with a polyester Dacron graft (Figure 4). The patient had collaterals to the azygos vein and thereby did not affect the hemodynamics while clamping the IVC.

Case 3

**Figure 5:**
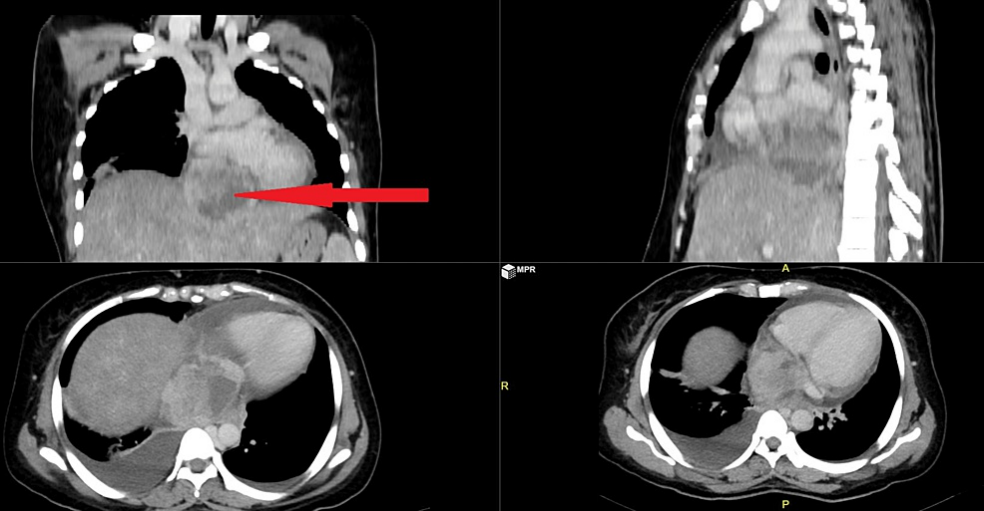
CT abdomen with the red arrow pointing to the supra-hepatic mass

A 30-year-old female presented with breathlessness and right shoulder pain. She was found to have a supra-hepatic tumor involving the IVC and extending up to the right atrium (Figure 5). The tumor was dissected away from surrounding tissues. Cardiopulmonary bypass was initiated with aorto-left femoral vein and superior vena cava cannulation before resecting the tumor. The IVC was opened, and the tumor was resected along with the mass.

## Discussion

Pearl was the first to describe an IVC LMS in 1871 [4]. Their incidence is more common in females and more often found in elderly women in their fifth to sixth decade. Inferior vena cava LMS are slow-growing tumors, and most of the patients remain asymptomatic, resulting in a delayed diagnosis. So these tumors have a poor prognosis, and more than half of them present with pulmonary metastasis. Symptoms are more often associated with the location of the tumor; segment I is located below the renal veins, and they usually present with lower limb edema, deep vein thrombosis, pain in the abdomen, and an abdominal mass. The region between the renal vein and the hepatic vein is classified under middle segment II, which represents 44% of the cases. These patients present with abdominal pain, nephrotic syndrome, and renal hypertension. Finally, the part between the hepatic vein and the right atrium is segment III, which represents 20% of the cases [5, 6]. Patients might present with Budd-Chiari syndrome and cardiac arrhythmias [7]. The overall prognosis for the middle segment tumors is better. The diagnosis of IVC LMS may be delayed as the tumor growth is very slow; 5% of these tumors manifest intraluminally, 62% present as extraluminal growth, and 33% of cases are both intraluminal and extraluminal [8].

Since the location of the tumor is variable, different surgical approaches and techniques should be applied based on tumor characteristics [9, 10]. The first two patients required midline laparotomies. The patient with a supra-hepatic tumor required a midline sternotomy to initiate a cardiopulmonary bypass. The first patient had an extraluminal growth, and a complete resection of the posterior wall of the IVC was done. Since the defect was small, primary repair of the posterior wall was possible. The second patient with sub-hepatic IVC LMS had intraluminal growth. There was no hypotension during clamping of the IVC for resection and reconstruction with a Dacron graft as the patient had collaterals from azygos. In the third patient, since the tumor was extending up to the IVC and right atrial junction, a cardiopulmonary bypass was initiated.

By revealing details regarding the genesis of the tumor and its connections to surrounding structures, CT and MRI scans play a critical role in the accurate diagnosis of LMS. These tumors can have areas of hemorrhage and necrosis and present as heterogeneous masses with peripheral enhancement. The only treatment that offers long-term survival is radical surgical resection, which involves removing the entire tumor [11]. Further information is gathered via contrast magnetic resonance venography and CT venography, respectively. These methods can show the location of the tumor, the degree of its invasion, and a complete longitudinal image of the mass [12]. The excision of an IVC LMS should be performed in specialized facilities by an experienced surgeon in order to avoid venous injury and substantial intraoperative bleeding.

We report these case series as each tumor was located in a different location and each had a different surgical approach. We advocate that chronic IVC tumors will have collateral, and clamping of the IVC for resection may not be detrimental. Tumors extending up to the right atrium (RA)-IVC junction may require cardiopulmonary bypass. Each tumor should be evaluated carefully, and the level of clamping should be such that it does not affect the collateral supply. If unprecedented, the patient may require a complete cardiopulmonary bypass for safe resection of the tumor.

## Conclusions

Inferior vena cava LMS are extremely rare tumors and their clinical manifestation may differ based on their location. Computed tomography aids in locating the tumor and in the planning of the surgery. A CT-guided biopsy is done to confirm the diagnosis, though it's rarely done. Complete surgical resection with a tumor-free margin offers a better prognosis, though chemotherapy can be used as an adjunct. Since the location of the tumor is variable, it requires a multidisciplinary approach involving surgical oncology and cardiothoracic and vascular surgeons.
